# Enhancing Quality of Life and Sexual Functioning in Female Androgenetic Alopecia: Therapeutic Potential of Hair Follicle-Derived Stem Cells

**DOI:** 10.3390/healthcare12060608

**Published:** 2024-03-07

**Authors:** Katarzyna Krefft-Trzciniecka, Hanna Cisoń, Alicja Pakiet, Danuta Nowicka, Jacek C. Szepietowski

**Affiliations:** Department of Dermatology, Venereology and Allergology, Wrocław Medical University, 50-368 Wrocław, Polandjacek.szepietowski@umw.edu.pl (J.C.S.)

**Keywords:** stem cell therapy, regenerative therapy, androgenetic alopecia, quality of life, female sexual function

## Abstract

Background: The study aimed to examine the impact of stem cell treatment on quality of life (QoL) and sexual functioning in women with androgenetic alopecia (AGA). Methods: Twenty-three women underwent a single session of autologous cellular micrografts (ACMs). The World Health Organization Quality of Life Brief Version (WHOQOL-BREF) and Female Sexual Function Index (FSFI) were used before and after 6 months. Results: The AGA severity decreased by an average of 1 point on the Ludwig scale (*p* = 0.004) after treatment. FSFI scores indicated sexual dysfunction in over half of the women at baseline, but they improved significantly post-treatment for arousal [median (IQR): 4.8 (1.5) vs. 5.10 (0.9); *p* = 0.035] and satisfaction [4.4 (1.4) vs. 4.8 (1.8); *p* = 0.025]. QoL scores improved after treatment in psychological health (57.96 ± 19.0 vs. 69.35 ± 14.0; *p* = 0.031) and environment (72.96 ± 13.4 vs. 81.09 ± 12.6; *p* = 0.007), but not in physical health and social relationships. No associations were found between the WHOQOL-BREF or FSFI domains versus age and AGA severity. Conclusions: AGA reduces QoL and impacts sexual functioning in women with AGA. The high treatment burden arises from the chronic and progressive nature of AGA, coupled with limited treatment effectiveness. Effective treatments for AGA, like ACM, are urgently needed to enhance patient-reported outcomes along with clinical results.

## 1. Introduction

Androgenetic alopecia (AGA) is a condition characterized by gradual and ongoing hair loss with an unpredictable pattern of progression [1]. A female pattern of hair loss can cause various psychological problems that reduce quality of life. The loss of self-confidence and decreases in self-esteem are common reactions to hair loss, especially in women [2]. The World Health Organization (WHO) explains that quality of life is a subjective assessment of the perception of reality through the lens of culture and the value system observed by its targets [3,4]. In European culture, physical appearance is highly valued, which is why AGA is such a significant problem [5].

In several studies, alopecia has been shown to have a psychosocial impact on both men and women, but the effects can be more severe and devastating on women [6]. Concerning the male population, the disease affects social functioning and emotional well-being, with pronounced detrimental effects observed among young men [7]. Camacho et al. [8] deduced that depression in the course of AGA was more common in women than in men and was most often described as a “minor”. Moreover, Russo et al. [9] concluded that women with AGA are characterized by greater social anxiety and social phobia compared to men with this disease. AGA is usually considered a benign process, mainly cosmetic, but numerous studies confirm that AGA can be a complication and manifestation of underlying systemic diseases, which further increases the patient’s anxiety [10]. Gonul et al. [2] examined and compared the quality of life in patients with AGA and alopecia areata using the Hairdex, an instrument developed to assess the quality of life in patients with hair loss. The results are very interesting and surprising, because, among people with AGA, hair loss had a more adverse impact on their emotions, functioning, and symptoms, compared to those with alopecia areata. A similar study was conducted by Jun et al. [11], who used the Hair Specific Skindex-29 to examine quality of life in patients with AGA and alopecia areata, identifying risk factors associated with its deterioration. Patients with AGA were more likely to report a symptomatic decline in quality of life (*p* = 0.033), while patients with alopecia areata showed significantly worse functional quality of life than AGA patients (*p* = 0.013).

Hair has been a significant aspect of identity and image for decades, holding sociological and psychological importance for a person’s appearance and personality [12,13]. Sexual health encompasses the physical, emotional, and mental well-being associated with sexuality, while impaired sexual health may have a significant impact on quality of life [14]. Sexual functions are closely linked to other aspects of behavior and cannot be considered independent phenomena. Sexuality has attracted considerable attention throughout human civilization and exerts a significant impact on people’s quality of life [13,15]. Quality of life and sexual health are multidimensional and share a bidirectional relationship.

AGA is a hair loss disorder mediated by dihydrotestosterone, which induces the miniaturization of hair follicles and transforms the final hair into vellus hair [16]. The pathogenesis of this condition also involves oxidative stress and microinflammation occurring around the hair follicles [17]. In women with AGA, hair loss can manifest clinically in a variety of ways. The two most common patterns are diffuse hair loss in the central front and posterior regions (Ludwig type—severity is evaluated on the Ludwig scale in three steps) or hair loss in the central part of the front scalp (Olson type), also known as front accentuation or “Christmas tree type” patterns [18,19,20]. Yu et al. [21] showed that the way people with AGA perceive their disease has a significant impact on health outcomes of other diseases. They also found that all patients considered alopecia to be a major concern with a considerable emotional impact. Patients reported relatively poor control over the disorder and its treatment, as well as a limited understanding of its course.

Minoxidil (topical administration) for female and male pattern hair loss and finasteride (oral administration) only for male pattern hair loss are the two drugs approved by the U.S. Food and Drug Administration (FDA) for the treatment of AGA [22]. Iamsumang et al. [23] reviewed studies involving oral and topical finasteride used in women. Although there are few studies available, their analysis concluded that finasteride was effective and safe for the treatment of female pattern hair loss. However, larger studies are needed to further validate these findings. The literature reports on the efficacy and safety of numerous other therapies, including platelet-rich plasma, dutasteride (systemic administration) (not approved by the FDA for alopecia), hair transplants, oral minoxidil, and low-level laser therapy [22].

Recently, regenerative medicine therapies have been gaining popularity. The goal of regenerative medicine is to restore the normal function of cells and organs by replacing and regenerating damaged cells or organs. Stem cells, cytokines, growth factors from either stem cells or hematopoietic tissues, and gene therapies are used for this purpose. Stem cells possess unique abilities such as self-renewal and the ability to exhibit anti-inflammatory and immunomodulatory effects. These characteristics make them applicable in the treatment of alopecia [24]. For the treatment of AGA, most reports focus on adipose-derived stem cells and hair follicle stem cells, while only a few studies are available on the use of bone marrow stem cells and Wharton’s jelly stem cells [25].

In this study, we aimed to investigate whether the stem cell treatment of AGA affects quality of life and sex life. Additionally, we sought to examine the correlation between sexual functioning and the quality of life among women with AGA after treatment.

## 2. Materials and Methods

### 2.1. Participants

This study was conducted through face-to-face surveys at the Clinic of Dermatology, Venerology and Allergology of Wroclaw Medical University, during which, participants completed questionnaires. Twenty-three female patients aged between 18 and 65 (mean 40 ± 12) years and diagnosed with AGA were included in the study.

Inclusion criteria were determined through clinical examination by dermatologists and assessed using the Ludwig scale. The distribution of the patients according to Ludwig’s female pattern hair loss classification showed that patients were classified as follows: Grade I—60.9%; Grade II—30.4%; and Grade III—8.8%. The primary exclusion criteria for this study included immunosuppression, cancer, severe chronic disease, pregnancy, breastfeeding, age under 18 years, hormonal contraception, hyperprolactinemia, hypothyroidism, active inflammation of the scalp, coagulation disorders, lignocaine allergy, and unstable emotional state. Patients with a positive antinuclear antibody (ANA) 3 test were also excluded. Additionally, patients who had received oral (finasteride, dutasteride, minoxidil, antiandrogens) or topical (minoxidil, prostaglandin analogs, corticosteroids) AGA-targeted treatment in the past 6 months were excluded. The study did not include patients who had used medical devices such as low-level laser therapy or undergone procedures such as platelet-rich plasma injections or micro-needling.

The patients received a single session of autologous cellular micrografting (ACM) obtained with the Regenera Activa^®^ device (Human Brain Wave SRL, Turin, Italy). Before and 6 months after the therapy, all participants completed two questionnaires: the World Health Organization Quality of Life Brief Version (WHOQOL-BREF) and the Female Sexual Function Index (FSFI). We used validated Polish versions of the FSFI and WHOQOL-BREF questionnaires. Both the FSFI and WHOQOL-BREF have a 4-week recall period. All participants provided written, informed consent prior to the enrollment and received instructions on how to complete the study questionnaires. The study was approved by the Bioethics Committee of Wroclaw Medical University (KB-1074/2021, approval date: 3 January 2022).

### 2.2. ACM Procedure

Under local anesthesia, five 2.5 mm diameter punch biopsies were collected from the scalp skin behind the patient’s ear. The collected samples were placed in medical devices (Class I, Rigneracons CE certified; Human Brain Wave SRL, Turin, Italy) and coated with 2 mL of sterile physiological solution. Then, the cell suspension was produced by turning the Rigeneracons at 80 RPM for 2 min. The suspension was then diluted with an additional 2 mL of sterile physiological solution. The resulting solution was injected into the scalp hair area using a 1 mL syringe and 30 mm needles. A volume of 0.1 mL was injected per point, with about 1 cm intervals between the needles.

### 2.3. Instruments

#### 2.3.1. World Health Organization Quality of Life Brief Version (WHOQOL-BREF)

The WHOQOL-BREF [26,27] is a self-report questionnaire that assesses quality of life in four domains: physical health (7 elements), psychological health (6 elements), social relationships (3 elements), and environment (8 elements). Each item is scored on a 1–5 Likert scale. After collecting filled questionnaires, the domain scores were transformed to a scale of 0 to 100, where higher scores indicate a higher quality of life. The recall period for this questionnaire is 4 weeks.

#### 2.3.2. Female Sexual Function Index (FSFI)

The FSFI is a 19-element measure, composed of six different areas of female sexual function, namely: desire (2 items), arousal (4 items), lubrication (4 items), orgasm (3 items), satisfaction (3 items), and pain (3 items). Total FSFI scores range from 2 (lowest possible score) to 36 (highest score) [28]. This scale is widely used as both a screening and outcome measurement tool for female sexual function, with a 4-week recall period [29]. The FSFI has a clinical cutoff score of 26.55 points total, as proposed by Wiegel et al. [30]. This score currently serves as the clinical standard for differentiating between patients with and without sexual dysfunction. In individual domains, scores below the median were considered indicative of dysfunction for that particular domain, i.e., for desire ≤ 3.6, for arousal ≤ 4.8, for lubrication ≤ 5.1, for orgasm ≤ 4.4, for satisfaction ≤ 4.4, and for pain ≤ 5.6.

### 2.4. Data Analysis

Variables with normal distributions were presented as mean ± standard deviation (SD), and those without normal distribution were presented as medians with the interquartile range (IQR) in parentheses. Box plots were used to illustrate the distribution of different domains of the WHOQOL-BREF and FSFI among patients. To investigate the differences in patients before and after treatment, paired *t*-tests (for WHOQOL-BREF domains) or Wilcoxon signed rank tests (for FSFI domains) were performed depending on the distribution of the variables, which was checked with the Shapiro–Wilk test. The correlation between WHOQOL-BREF and FSFI was assessed with Spearman’s correlation coefficients. The effect size was calculated as described by Cohen [31]. For variables that underwent a paired *t*-test with Cohen’s d statistic, d = (M_1_ − M_2_)/SD_pooled_, where M_1_ and M_2_ are the means of the compared groups and SD_pooled_ is a pooled standard deviation of the two groups (SD_pooled_ = √[(SD_1_^2^ + SD_2_^2^)/2]. For variables that underwent Wilcoxon signed rank tests, effect size was calculated with r, defined as r = z/√n, where z is a z-score and n is a sum of the grouped sizes. All calculations were performed using SigmaPlot 14.5 (Systat, Software Inc., San Jose, CA, USA).

## 3. Results

The results included data from all 23 patients with AGA. Table 1 presents the characteristics of the study patients. Following the ACM treatment, the severity of AGA significantly decreased by an average of 1 point on the Ludwig scale (*p* = 0.004). We did not detect any association between the WHOQOL-BREF or FSFI domains and age or AGA severity.

**Figure 1:**
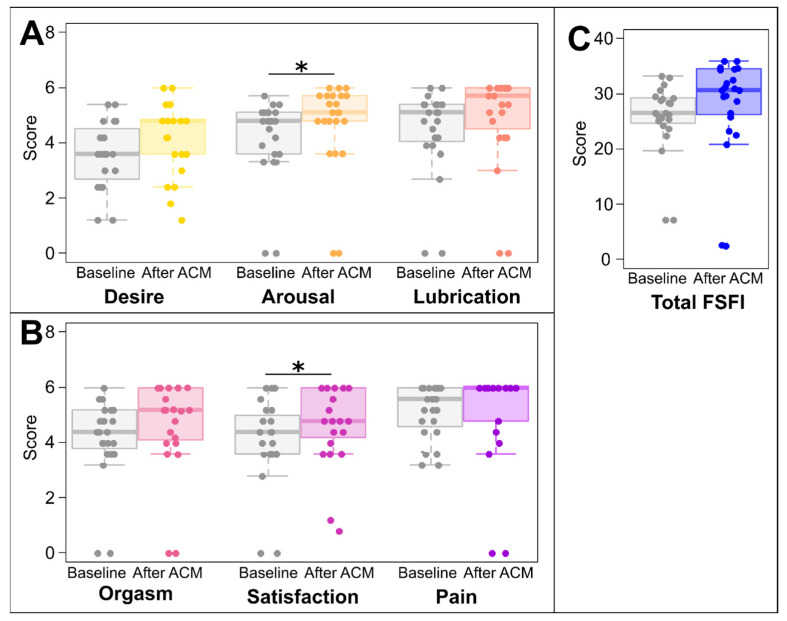
Female Sexual Satisfaction Index scores. Domains (**A**,**B**) and total score (**C**). * *p* < 0.05; values from Wilcoxon Signed Rank test; ACM, autologous cellular micrograft procedure.

Before treatment, 11 patients had scores below 26.55, indicating sexual dysfunction, and this number decreased to 6 patients with sexual dysfunction after 6 months following the ACM session. At baseline, the majority of the patients experienced sexual dysfunction in specific domains, with 65% of patients reporting below the median for desire and arousal, 61% below the median in lubrication and pain, and 57% below the median with regard to orgasm and satisfaction. The FSFI results are summarized in Figure 1. After ACM treatment, patients reported significantly higher arousal with a median of 4.8 (1.5) before and 5.10 (0.9) after treatment (*p* = 0.035, effect size r = −0.31, indicating medium effect), as well as satisfaction with a median of 4.4 (1.4) before and 4.8 (1.8) after treatment (*p* = 0.025, effect size r = −0.324) as measured by the FSFI. The total FSFI score, as well as the desire, lubrication, orgasm, and pain indexes, did not differ significantly between study points.

**Figure 2:**
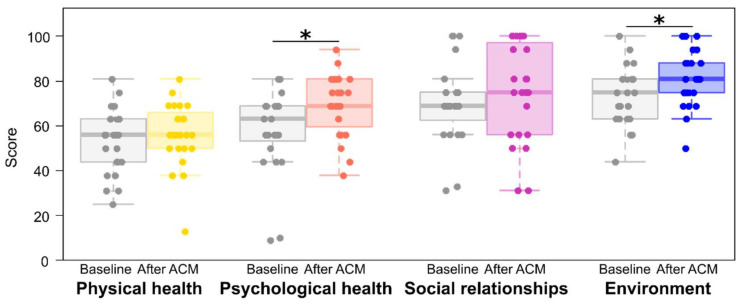
WHOQOL-BREF scores. * *p* < 0.05 from two-tailed *t* test; ACM, autologous cellular micrograft procedure.

Among the WHOQOL-BREF domains (summarized in Figure 2), the AGA patients reported the lowest quality of life in the physical health domain. The assessment with WHOQOL-BREF showed that 6 months after the ACM procedure, the patients experienced higher quality of life in psychological health (mean before 57.96 ± 19.0 vs. mean after 69.35 ± 14.0; *p* = 0.031, the effect size measured by Cohen’s d was d = 0.68, indicating a medium effect) and environment (mean before 72.96 ± 13.4 vs. mean after 81.09 ± 12.6; *p* = 0.007, Cohen’s d was 0.63). There were no significant changes in reported physical health and social relationships.

The analysis of correlations between WHOQOL-BREF scores and FSFI results revealed that, across all domains, overall quality of life and sexual health were moderately to strongly positively correlated (Table 2). The strongest associations (above 0.7 Spearman’s rho) were found between FSFI arousal and social relationships and FSFI satisfaction and social relationships.

## 4. Discussion

Our analyses indicated that female patients with AGA had a reduced quality of life in the physical health domain, which, however, was not dependent on ACM treatment. The applied therapy significantly improved patients’ quality of life in the mental health and environmental domains.

Researchers emphasize that women with AGA experience reduced quality of life, a finding consistent with our study. Moorthy et al. [32] also employed the WHO-BREF scale to assess quality of life, using a similar approach employed in our study. Additionally, they used the Hairdex questionnaire, a hair- and scalp-specific measurement tool designed to assess the specific effects of hair loss on the quality of life of patients. This questionnaire consists of 48 items categorized into five domains: symptoms, functions, emotions, self-confidence, and stigma. Their study included 170 patients with AGA of both sexes. The study found that in women under 30, the physical health and mental health domains were most impaired. Among singles, the symptom and emotion domains were affected, while those with less education exhibited impairment in all domains except physical health. Another study conducted by Reid et al. [33] discovered that patients often view their hair loss as more distressing in terms of quality of life than dermatologists perceive it to be. While physician and patient ratings of clinical severity align, the study found that clinical severity alone does not predict the impact of the disease on a patient’s quality of life, hence the importance of employing additional validated tools to assess quality of life and using advanced devices to monitor disease progression. In a recent systematic review, Aukerman and Jafferany [5] investigated the psychosocial consequences of AGA and concluded that female patients experience severe dissatisfaction with their hair, leading to a decline in overall body image. Furthermore, dissatisfaction with one’s body image significantly contributes to reduced desire and arousal [34]. In the present study, desire was the most affected area of sexual function at baseline, and it did not improve after therapy. However, we observed significant improvements in the domains of arousal and satisfaction, suggesting that ACM treatment has a positive effect on the sexual health of female AGA patients. These two FSFI domains exhibited the strongest association with the social relationships domain of WHOQOL-BREF. The latter did show some improvement after ACM treatment, although it was not statistically significant. Van der Donk et al. [35], based on their study on a group of 58 women, concluded that women with AGA often feel less attractive. Positive perceptions of one’s attractiveness are associated with higher sexual satisfaction, higher sexual frequency, and more sexual partners [36]. Improving AGA through treatment and enhancing patients’ appearance can boost their self-esteem, thereby influencing their sex lives. Our analysis of the associations between sexual health scores and quality of life indices also suggests that improvements in physical, and psychological health, as well as social relationships and environmental factors, are all linked with better sexual function. Biondo et al. [37], similarly to Reid et al. [33], hypothesized that women with AGA often rate their condition as more severe than their physicians do. However, their study, which examined disparities in perceptions of hair loss severity between patients and clinicians, revealed that women seeking treatment for AGA often underestimate the severity of their disease. Therefore, the findings of Biondo et al. suggest that it can be worthwhile to raise awareness among patients about alopecia and initiate effective treatment, as untreated AGA can progress and negatively affect both quality of life and sexual function. Tas et al. [38] used the ASEX scale, a tool designed to assess the sexual functioning of patients with AGA according to the severity of the disease. This scale assesses five domains: drive, arousal, penile erection/vaginal lubrication, ability to reach orgasm, and orgasmic satisfaction. Their analysis showed that the likelihood of patients developing psychosexual disorders increases with disease progression. Consequently, patients with AGA, especially those in advanced stages, should be referred to psychological or sexology counseling. While mental status becomes improved, the pain domain of the FSFI does not change in response to AGA treatment. Dyspareunia, defined as pain during sexual intercourse, has a complex etiology [39]. Leeners et al. [40] showed that dyspareunia often occurs alongside other psychiatric disorders, with depression being the most common. Depression is an illness frequently associated with AGA, implying a possible overlap of these conditions within our study group.

The availability of studies examining the effect of a specific therapeutic method in relation to the quality of life in women with AGA is limited. While most studies have focused on quality of life in the AGA population, it seems interesting to explore the impact of specific treatments on both quality of life and sexual functioning. Due to limited FDA-approved treatments, experimental and off-label treatments are commonly explored for this condition.

Pharmacotherapy is often used for treating AGA in women; however, many of the agents have not been approved by the FDA for this specific indication. Zhuang et al. [41] conducted a study involving 31 patients diagnosed with AGA to assess the impact on quality of life and clinical improvement after 12 months of using 2% topical minoxidil. They used the Visual Analog Scale (VAS) and the Dermatologic Quality of Life Index (DLQI). The results indicated that hair loss is significantly associated with a reduced quality of life, and the use of topical minoxidil was observed to improve this condition. In another study, Yamazaki et al. [42] investigated how 1 mg of oral finasteride taken for 6 months affected the quality of life of 27 men with AGA aged between 19 and 76 years. They used WHOQOL-BREF, the DLQI, the State–Trait Anxiety Inventory (STAI), and VAS scales. The comparison of WHOQOL-BREF scores before and after the administration of 1 mg of finasteride showed an overall improvement in the quality of life in the study group, although the improvement in individual domains was not statistically significant. It is worth noting that while finasteride treatment is well established in men, only a small number of studies focus on the use of 5 α-reductase inhibitors in female pattern hair loss, which is considered as an off-label treatment. Seale et al. [43] emphasized the potential side effects that 5 α-reductase inhibitors may cause in women when used as an oral treatment for female pattern hair loss and frontal fibrosing alopecia. However, the frequency of sexual function impairment after the administration of dutasteride or finasteride in the identified studies varied. Two studies reported that the daily administration of 5 mg of finasteride contributed to treatment discontinuation due to decreased libido. The occurrence of post-finasteride syndrome in women was not determined in that review. However, post-finasteride syndrome, which includes, among other adverse side effects, sexual dysfunction secondary to 5 α-reductase inhibitors, is well described among male patients and includes the loss of libido and erectile dysfunction [44]. Although finasteride is not yet FDA-approved for female patients, its potential as an alternative treatment is being considered [23]. To date, the limited data available seem to indicate very few side effects of 5 α-reductase inhibitors on sexual function in women [43]. To our knowledge, there are no published studies evaluating the effect of AGA treatment on women’s sexual functioning, and the available data on the association of AGA and impaired sexual function are contradictory. Sancak et al. [14] evaluated the correlations between female sexual dysfunction and AGA in premenopausal women, finding that the presence of AGA significantly impaired their sexual function. Conversely, Eyada et al. [45] found no correlation between the incidence of sexual function impairment and AGA.

Topical preparations for AGA are characterized by a lower frequency of side effects, but their high treatment burden limits patient adherence and, consequently, effectiveness. In a study by Zac and da Costa [46], quality of life, treatment satisfaction, and dermatoscopic criteria were evaluated among female patients with AGA undergoing treatment with topical 5% minoxidil. The study concluded that treatment with 5% minoxidil had positive psychological benefits. Interestingly, patient satisfaction and quality of life were not found to be related to age, alopecia severity, or hair shaft reduction per follicle unit. Another study conducted by Moorthy et al. [32] on 170 women with AGA showed that female AGA was associated with significantly reduced quality of life, particularly among young, less educated, and single women. This suggests that the potential benefits of treatment could be age-dependent. In our analysis, we considered the association between age and found no correlation between improvements in quality of life after treatment with hair follicle stem cells and the age of the patients. Vastarella et al. [47] retrospectively studied the effect of oral minoxidil at a dose of 0.5 mg to 2 mg daily in women diagnosed with AGA. The study group consisted of 12 women with AGA (Ludwig scale I-3-III) with a mean age of 36.7 ± 18.8 years. The Women with Androgenetic Alopecia Quality of Life Questionnaire (WAA-QoL) was used to assess quality of life, showing a statistically significant improvement after 24 weeks compared to baseline. The median WAA-QoL score was 70.33 at baseline and decreased to 25.58 at 24 weeks (*p* < 0.01).

There are only a few studies that have investigated quality of life among women with AGA treated with platelet-rich plasma. Meyers et al. [48] conducted a study examining the effect of platelet-rich plasma treatments on the quality of life of 92 female and male patients with pattern hair loss. Using the Hairdex 48 scale, they found that this type of treatment is effective in improving the overall quality of life. The study also revealed a decrease in the domains of symptoms, emotions, and functioning, with improvements demonstrated in the domains of stigma and self-confidence. A study conducted by Bruce et al. [49] was a randomized, controlled pilot trial investigating the efficacy of platelet-rich plasma in comparison to topical minoxidil foam for the treatment of AGA among women. The secondary outcome of this study included the measurement of quality of life using a 16-item questionnaire administered at baseline and after the 12-week treatment period. Notably, no improvements were observed after minoxidil treatment, whereas the platelet-rich plasma group demonstrated significant improvement in aspects such as self-consciousness about people looking at their hair, feelings of jealousy, ability to socialize with people, and satisfaction with overall hair appearance.

Hair transplants involve the surgical transplantation of hair follicles from a donor area, typically the back or sides of the scalp, to areas with hair loss. This procedure is invasive and expensive but effective, providing natural-looking results. Nilforoushzadeh et al. [50] conducted a survey involving 35 men to assess quality of life and self-esteem before and after hair transplant surgery. They used the Rosenberg Self-Esteem Scale (RSES) and the DLQI and demonstrated a statistically significant difference in self-esteem and quality of life before and after the restoration surgery. Additionally, the study revealed a statistically significant relationship between educational achievement and quality of life. This study suggests that as self-esteem improves, patients’ quality of life also improves, emphasizing the close connection between self-esteem and a satisfying physical appearance. Xiao et al. [51] investigated health utility measures among patients with AGA after a hair transplant. They compared 31 patients with alopecia with 237 otherwise healthy people. After the hair transplant, the improvement in time trade-off—a technique used to measure the experienced quality of life for economic analyses—was significantly greater for patients with AGA (+0.08 ± 0.12 vs. +0.02 ± 0.09; *p* = 0.0070) and significantly more often exceeded the minimal clinically important difference (45.2% vs. 16.9%; *p* = 0.0006).

A limitation of our study is undoubtedly the small sample size and the lack of a placebo group. A major challenge is to find women with AGA who are willing to abstain from other types of treatment for 6 months. In the future, we aim to investigate how stem cell therapy affects quality of life and sexual functioning in men affected by the disease. It also seems interesting to compare this therapy with others, especially with FDA-approved drugs, in terms of their impact on quality of life. Another important limitation of our study is that the quality of life of our female patients may be affected by factors other than AGA, which were not controlled for. We lacked sufficient information to account for factors that could influence quality of life, such as socialized frequency and marital status. Certainly, collaboration with psychiatrists and psychotherapists, who would be able to professionally assess the mental state of the patients, could have ruled out other potential causes of lowered mood or dissatisfaction with sexual life. Another limitation of our work is the inability to interpret the interesting and surprising result of improved quality of life in the environmental domain. Unfortunately, due to the lack of information about the demographics of the patients, we were not able to interpret its reliably. This area requires further research.

## 5. Conclusions

Dermatologists need to recognize the tremendous impact that AGA has on the quality of life and sexual functioning of women affected by the condition. The assessment of quality of life and sexual functioning in women with AGA is increasingly important for assessing the effects of the disease on patients and the required treatment. The chronic and progressive nature of AGA, coupled with limited treatment effectiveness, contributes to substantial suffering for those affected. Therefore, there is a pressing need for novel and effective treatments for AGA. Highlighting the potential advantages of a multidisciplinary approach that includes dermatologists, endocrinologists, and psychologists, this study offers optimism regarding the ability of new stem cell treatments to diminish the severity of AGA. This, in turn, holds the promise of enhancing the overall quality of life for individuals affected by the condition.

## Figures and Tables

**Table 1:** Age and androgenetic alopecia severity in patients.

Age	N	Mean (SD)
<30 years	9	28 (2)
>30 years	14	47 (10)
**Androgenetic alopecia severity**	**N Before**	**N After**
Ludwig score 1	7	15
Ludwig score 2	14	7
Ludwig score 3	2	1

**Table 2:** Correlations between sexual health and quality and life domains.

	Physical Health	Psychological Health	Social Relationships	Environment
Desire	0.26	0.46 *	0.56 **	0.28
Arousal	0.53 **	0.48 **	0.71 **	0.52 **
Lubrication	0.34 *	0.55 **	0.69 **	0.47 **
Orgasm	0.48 **	0.47 *	0.63 **	0.40 *
Satisfaction	0.54 **	0.46 *	0.76 **	0.35 *
Pain	0.33 *	0.52 **	0.59 **	0.44 *

Shown are Spearman’s correlation coefficients. * *p* < 0.05, ** *p* < 0.001.

## Data Availability

The data that support the findings of this study are available on request from the corresponding author.
